# Mortality During In-Hospital Stay and the First 24 h After Decompressive Craniectomy in Severe Traumatic Brain Injury: A Multi-Center, Retrospective Propensity Score-Matched Study

**DOI:** 10.3390/jcm14155540

**Published:** 2025-08-06

**Authors:** Thomas Kapapa, Martin Petkov, Andrej Pala, Dieter Woischneck, Franziska Schiller, Stefanie Jesuthasan, Frederike Schiller, Hendrik Bracht, Benjamin Mayer, Marcel Oehmichen

**Affiliations:** 1 Department of Neurosurgery, University Hospital Ulm, Albert-Einstein-Allee 23, 89081 Ulm, Germany; 2 Department of Neurosurgery, Hospital Landshut, Robert-Koch-Strasse 1, 84034 Landshut, Germany; 3 Department for Anaesthesiology, Intensive, Emergency, Transfusion Medicine and Pain Therapies, Bielefeld University Hospital, House Gilead I, Burgsteig 13, 33617 Bielefeld, Germany; 4 Institute for Epidemiology and Medical Biometry, University of Ulm, Schwabstrasse 13, 89075 Ulm, Germany; 5 Military Hospital Ulm, Department of Neurosurgery, Oberer Eselsberg 40, 89081 Ulm, Germany

**Keywords:** intracranial pressure, brain edema, survival, outcome, morbidity, intensive care, aging, traumatic brain injury, secondary brain damage

## Abstract

**Objectives**: Early death after trauma has been described several times. Little is known about it after traumatic brain injury (TBI) and decompressive craniectomy (DC). The aim of this study was to characterize patients who die after a TBI and DC during their in-hospital stay. **Methods**: In a subgroup analysis of a retrospective, multicenter, and observational study, non-survivors from in-hospital stays treated for severe TBI and DC were included. Propensity score matching (PSM) was used. **Results**: A total of 223 patients with severe TBI were treated with DC, and there were 65 (29.1%) patients who did not survive. Of these, 22 (33.8%) died within the first 24 h. Non-survivors were older (*p* = 0.010), and pupillomotor dysfunction and a higher heart rate on admission were more common (*p* < 0.001). PSM patients for overall survival (41, 18.4%) differed in mean heart rate from the deceased (*p* = 0.030). In a multivariate model, age (OR: 1.045, *p* = 0.013, CI95%: 1.010 to 1.082), Quick value (OR: 0.965, *p* = 0.049, CI95%: 0.931 to 1.000), and heart rate (OR: 1.099, *p* = 0.030, CI95%: 1.009 to 1.197) were confirmed as predictive factors. **Conclusions**: Even after DC, known factors, such as chronological age and comorbidities, have a significant influence on mortality. The value of DC in an aging society for a particular severity of TBI should be further assessed on the basis of prospective studies.

## 1. Introduction

Much has been written about the outcome after trauma in general and about the causes of early death after trauma [1,2,3]. In the literature, the term early death is often used to describe a period of 24 h after the trauma and includes death immediately after the trauma and during the first treatment in hospital [4]. However, very little is known about early death following traumatic brain injury (TBI) and decompressive craniectomy (DC), i.e., after stabilization of the patient and surgical intervention for intracranial pathology. The outcome after DC and severe TBI depends on patient selection and on optimizing the timing of DC [5]. The indications and timing for a DC can be roughly categorized into primary (after initial evacuation of mass lesions) and secondary categories (in refractory intracranial hypertension initially conservatively treated) [6,7,8]. The mortality rate of severe TBI is reported to be between 20 and 30 percent [9], while after severe TBI and DC it is between 19 and 58 percent [5,10,11,12,13,14,15,16,17,18,19,20,21]. Studies that assess and discuss in-hospital mortality during the in-hospital stay or mortality in the first 24 months after TBI and DC are very rare. In addition, death during first (or primary) in-hospital stay must be considered separately from death during secondary hospitalisation, such as subsequent (early) rehabilitation or readmission for other clinical complications, such as pneumonia or myocardial infarction, as the latter have no direct link to the effect of trauma. The decision to focus on 24-h mortality is based on both clinical and methodological considerations. The first 24 h following severe TBI represent a critical window during which the majority of early deaths occur, often due to the direct impact of trauma [22]. Moreover, this time frame reflects the immediate effectiveness of pre-hospital and early in-hospital care, including airway management, hemodynamic stabilization, neurosurgical intervention, and intensive monitoring [22,23,24]. From a clinical perspective, deaths within 24 h are often non-survivable or minimally modifiable, but identifying these cases helps to distinguish between irreversible injury and delays or deficits in acute care.

The mortality rate from primary hospitalisation (primary in-hospital stay) after DC in TBI is described as being between 12 and 43 percent [9,12,25,26,27,28]. Consistent with the fact that there are different temporal needs for DC, it stands to reason that there are subpopulations of patients who die during the primary in-hospital stay after TBI and DC [20,29,30]. Therefore, the aim of this study is to characterize the patients who do not survive the primary in-hospital stay and to further describe the patients who die in the first 24 h of the in-hospital stay. This knowledge could improve predictive assessment and further clinical management.

## 2. Materials and Methods

### 2.1. Study Design

This study reflects a post hoc analysis of a retrospective, multicenter, and observational study. Patients were recruited at three large independent neurosurgery departments. Each neurosurgery department provides treatment in a tertiary care concept and has access to a neuro-intensive care unit. Patients were either primarily admitted or treated as secondary transfers. The treatment algorithms in the departments, which did not differ, were based on the national and international guidelines for the treatment of severe TBI and in accordance with the recommendation for DC after TBI [31,32,33,34]. The study was conducted in accordance with the Helsinki Declaration [35] and was reviewed and approved several times by the Ethics Committee of the University of Ulm, Germany (number 439/17 on 13 December 2017 and number 63/23 on 23 March 2023). This ethics committee granted a waiver for obtaining individual consent for this study, as it was a retrospectively conducted.

### 2.2. Patients

The study included patients consecutively treated for severe TBI and DC between January 2005 and December 2022. Severe TBI was defined by the clinical condition and assessment of consciousness according to the Glasgow Coma Scale Score ≤ 9 [30,36] and the Marshall computed tomography (CT) grading score > 2 [37] at first contact (emergency doctor) and/or on admission. There were no restrictions in terms of chronological age. Patients in whom no DC was performed or in which data about the severity of the TBI or initial status of consciousness were not available were excluded.

### 2.3. Primary In-Hospital Stay (Primary Hospitalisation)

In contrast to other studies, this study only considers the period of the first inpatient treatment after the TBI in one of the described neurosurgical departments. This period is defined as the primary in-hospital stay. Patients are often transferred to an (early) rehabilitation hospital after acute treatment of TBI. In the case of intracranial complications, such as hydrocephalus or cranioplasty, but also extracranial complications, such as myocardial infarction or the use of renal replacement procedures, patients may be transferred back to the primary care hospital. For the authors, this re-admission represents a secondary hospitalisation. This is not the subject of this study.

### 2.4. Procedures

Neurocritical care treatment was carried out in accordance with current national and international guidelines [31,32,33]. The indication for DC (fronto-parieto-occipital with temporal enlargement) corresponds to international recommendations and an escalating therapy algorithm [34]. In brief, the standard operating procedures allowed for a distinction between primary and secondary DC. Primary DC was performed shortly after the patient’s admission to hospital, following imaging of an intracranial mass, such as an acute subdural hematoma, intraparenchymal hematoma or, more rarely, an extradural hematoma, as a decision not to reimplant the bone flap at the end of the mass evacuation [34]. Secondary DC is a step in the escalating therapy algorithm. It was used in cases of therapy-resistant increased intracranial pressure (ICP) and/or continuously decreasing neurological status (GCS < 9) despite the exhaustion of all recommended conservative measures. Escalating non-surgical management was initiated at an ICP > 20 mmHg. Refractory elevated ICP was defined as a value > 25 mmHg for 15 min without decreasing despite interventions, such as positioning, body temperature normothermia (36 to ≤37.5° Celsius), deepening of sedation, hyperventilation, or hyperosmolar solutions, such as mannitol or 10% NaCl [31,32,33]. The dimensions of the DC have already been published and correspond to the recommendations [34]. Conservative ICP therapy was continued during the postoperative treatment phase in the intensive care unit.

### 2.5. Assessments and Data Collection

To identify the patients, analog and digital patient archives were searched according to the ICD10 codes S00 to S09, with a particular emphasis on code S06. The documentation of the surgeries performed during this period was also searched in order to identify the DC procedure and assign it to a severe TBI. Demographic, pre-hospital, in-hospital, and post-hospital data were collected. Unfortunately, there were significant gaps in the documentation of the pre-hospital phase (e.g., first GCS score or first blood pressure and pulse), so they could not be used for statistical analysis. Therefore, the first GCS score, first blood pressure, and pulse on arrival in the emergency room were used. Initial hospital laboratory results were calculated for glucose in mg/dL, partial thrombin time (PTT) in seconds, platelet count in giga/L, fibrinogen in mg/dL, leucocytes per µL, hemoglobin (Hb) in g/dL and hematocrit in percent. The initial CT scan was reviewed by two different neuroradiologists and four different neurosurgeons to assess the degree of Marshall injury score. Additional injuries to the thorax, abdomen, extremities and spine were only included in the analysis if they had conservative or surgical consequences. Surgical consequences were, for example, the placement of a chest drain or the application of an external fixation. Conservative consequences were, for example, the application of a fracture splint or the detection of lung contusions on CT, which resulted in a special ventilation regime.

### 2.6. Outcome Measures

The aim of this study was to describe the cohort of patients who did not survive the primary in-hospital treatment phase. Therefore, survival (outcome) of the first treatment phase was defined as the first endpoint (death during the primary in-hospital stay versus survival and discharge or transferred from the primary in-hospital stay). However, there were several patients who did not survive the first 24 h of the primary in-hospital stay. Again, we made a distinction in the outcome or in the definition of the endpoint as a secondary endpoint: death within the first 24 h of the primary in-hospital stay versus survivors and those discharged or transferred from the primary in-hospital stay.

### 2.7. Statistical Analysis

Primarily, the cohort of patients who did not survive primary in-hospital treatment was compared with the cohort of patients who were discharged alive. Descriptive statistics were used to describe and summarize data. The mean value with standard deviation and the median with range are used for this purpose. Mean values and standard deviations as well as median values and their range are also given for the GCS value and the Marshall Score. The authors are aware that the GCS and Marshall Score are ordinal values and that the mean is not the correct statistical expression for the center of the values. However, mean values are given in many publications, so the authors decided to give the mean and median for better comparison. Non-parametric tests, such as the Kruskal–Wallis H or Mann–Whitney U tests on one side, and the t-test, ANOVA, chi-square test, Fisher’s exact test, and regression analysis on the other, were used to apply inferential statistical methods in order to draw conclusions about the whole population. The assumption of the normal distribution of the values was checked using the Kolmogorov–Smirnoff test [38]. Binary logistic regression was used for the simple comparison of deceased and survivors. Due to the fact that the propensity matching and the resulting case–control pairs in the data created a manually generated dependency, the formal requirement was met by analyzing the data using conditional logistic regression (Cox regression). In selected cases, the results are presented in graphs and tables. The Graph Pad Prism 10.2.3 software (Dotatics, Boston, MA, USA) was used to create the graphs. It was also used to visualize statistical results. However, the statistical methods in the graphs differed from the methods used in SPSS 28 (IBM, Armok, ID, USA), so that the statistical results in the graphs were given for both methods.

Compared to unadjusted group comparisons, we used propensity score matching (PSM) because it offers several methodological advantages. It reduces confounding by balancing observed covariates between groups, thereby mitigating selection bias and approximating the conditions of a randomized trial. This allows for more robust estimation of treatment effects in observational data, especially when randomization is not feasible. For the one-to-one propensity score matching function, SPSS 28 was used [39]. Patients who did not survive the primary in-hospital treatment were matched with similar patients who did survive. We used a matching tolerance of 0.05, restricting the distance between the propensity scores of two participants to be matched to 0.05 SDs of the logit of the propensity score [40]. In a second step, we repeated this propensity matching again for patients who died within the first 24 h after treatment. These were matched to patients who had survived the primary stay. As a very small group of the deceased was compared to a larger group of survivors, the matching tolerance was set at 0.2. The 0.2 tolerance limit recommended in the literature is not exceeded [41]. The reason for this very narrow tolerance range was the attempt to identify very identical patient pairs of deceased patients and survivors. Unfortunately, this was at the expense of the group size of the matched patients. However, this limitation was accepted in order to better characterize the patients. Matching variables were defined as chronological age, sex, Glasgow Coma Scale Score at admission, pupil function disturbance, and Marshall score on first imaging. The significance level was set at *p* ≤ 0.05, whereas all *p*-values are interpreted in an exploratory manner only.

## 3. Results

During the mentioned period, 223 patients with severe TBI were treated with DC. The mean age was 49.9 (SD: 21.38) years and the cohort was approximately 30% female. The mean time between trauma and surgery was 28.8 (SD: 81.48) h. However, there was one patient who required surgery 936 h after trauma. Excluding this patient, the mean time was 23.8 (SD: 45.02) h. The number of patients with pupillomotor dysfunction was 58 (26%) and the median GCS score was 3 (3–15) in the emergency department. The median Marshall score was 4 (1–6). Information on clinical characteristics, the initial vital signs, and additional injuries can be found in Table 1.

**Figure 1:**
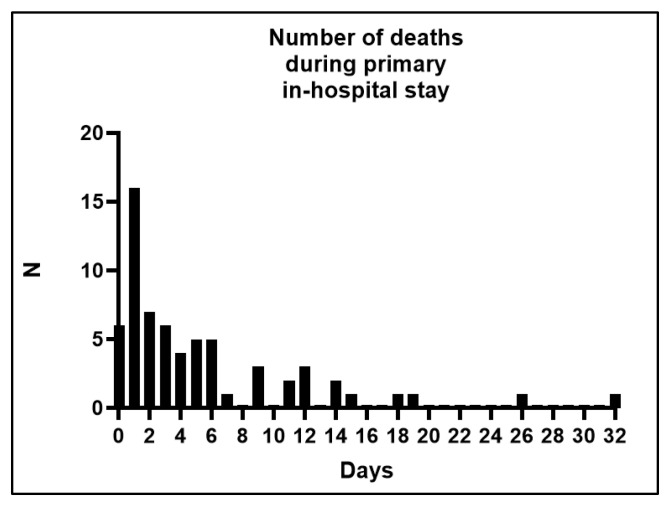
Number of deaths during the primary in-hospital stay.

There were 65 (29.1%) patients who did not survive primary in-hospital therapy. A total of 22 (32.3%) of these 65 patients died in the first 24 h of in-hospital therapy (Figure 1).

**Figure 2:**
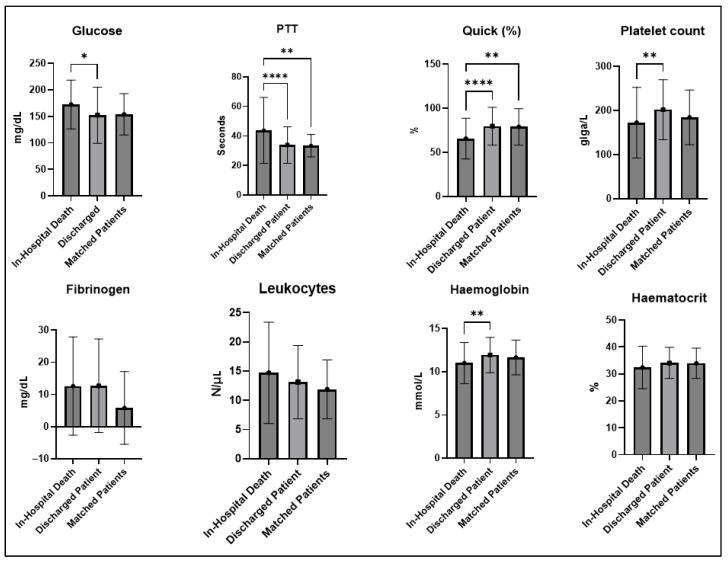
Results of the first laboratory tests after admission in non-survivors and survivors, and propensity-matched survivors (* = *p* <0.05, ** = *p* < 0.01, **** = *p* < 0.0001).

With a mean of 55.2 (SD: 22.23) years, they were significantly older than 158 (70.9%) patients who survived (47.7 years, SD: 20.69) (*p* = 0.010, Mann–Whitney U test). Pupillomotor dysfunction was significantly more common in the deceased (63.8% versus 37.3%, *p* < 0.001, Fisher’s exact test) and the heart rate on admission was significantly higher in the deceased (95.2, SD: 32.37) compared to the survivors (82.0, SD: 11.69) (*p* < 0.001, Mann–Whitney U test). There was no significant difference in sex distribution, time between trauma and surgery, Glasgow Coma Scale score at admission, Marshall score, mean arterial pressure (MAP), and incidence of additional injuries (Table 1). With regard to the laboratory test results on admission, there were only significant differences between the deceased and the survivors with regard to glucose (*p* = 0.002, Mann–Whitney U test), PTT (*p* < 0.001, Mann–Whitney U test), Quick value (*p* < 0.001 Mann–Whitney U test), the platelet count (*p* < 0.001 Mann–Whitney U test), and hemoglobin (*p* = 0.008, Mann–Whitney U test) (Figure 2).

Propensity score matching revealed 41 (18.4%) patients who survived the first treatment phase who matched the deceased (standardized mean differences are given in Supplementary Table S1). These patients differed significantly in mean heart rate (95.2, SD: 32.37 versus 84.9, SD: 17.23, *p* = 0.030, Mann–Whitney U test). There were no significant differences in the incidence of additional extracranial injuries or MAP (Table 1). In the comparison of laboratory tests between the deceased and the propensity score-matched patients, the only significant differences were found in PTT (*p* = 0.006, Mann–Whitney U test) and Quick values (*p* = 0.002, Mann–Whitney U test) (Figure 2).

There were 22 (9.9%) patients who died within the first 24 h of treatment. With a mean age of 57.1 (SD: 24.65) years, they were significantly older than patients who were discharged (47.7, SD: 20.69) (*p* = 0.036, Mann–Whitney U test). They underwent surgery significantly earlier than discharged patients (median (range) time between trauma and surgery 4 (1–29) h versus 7 (1–936) h; *p* = 0.033 Mann–Whitney U test) and had significantly more frequent pupillary dysfunction (76.2% versus 37.3%, *p* < 0.001, Fisher’s exact test). Their mean heart rate was 103.3 (SD: 25.84), which was significantly higher than that of discharged patients (82.0, SD: 19.68) (*p* < 0.001, Mann–Whitney U test). Additional thoracic (*p* = 0.041) and abdominal (*p* = 0.025) injuries were more common in the deceased (Table 2).

**Figure 3:**
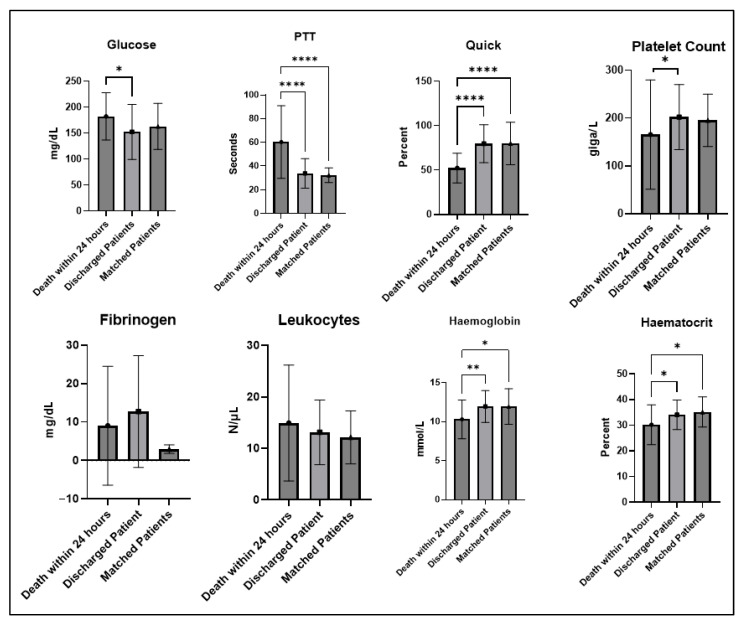
Results of first laboratory tests after admission in 24 h non-survivors, survivors, and propensity-matched survivors (* = *p* <0.05, ** = *p* < 0.01, **** = *p* < 0.0001).

Propensity score matching resulted in 21 (9.4%) matching patients who survived primary therapy. They only differed significantly from the deceased patients with a lower heart rate (103.3, SD: 25.84 versus 76.7, SD: 14.08) (*p* < 0.001, Mann–Whitney U test) (Table 2). A comparison of the laboratory test results on admission between the patients who died within the first 24 h, the survivors, and the propensity score-matched survivors revealed significant differences with regard to glucose (*p* = 0.0339, Mann–Whitney U test), PTT (*p* < 0.001, Mann–Whitney U test), Quick value (*p* < 0.001 Mann–Whitney U test), platelet count (*p* < 0.022 Mann–Whitney U test), hemoglobin (*p* = 0.029, Mann–Whitney U test), and hematocrit (*p* = 0.012, Mann–Whitney U test) (Figure 3).

In the comparison of deceased and survivors, univariate significant effects were found for the chronological age, time between trauma and surgery, pupillary dysfunction, heart rate, glucose, PTT, Quick value, platelet count, and hemoglobin laboratory values. In a multivariate model, only age (OR: 1.045, *p* = 0.013, CI95%: 1.010 to 1.082) and Quick value (OR: 0.965, *p* = 0.049, CI95%: 0.931 to 1.000) could be confirmed (Table 3).

For the comparison of patients who died within the 24 h after admission and survivors, the variables of chronological age, additional thorax and abdominal injuries, pupillary dysfunction, heart rate, glucose, PTT, Quick value, platelet count, and hemoglobin and hematocrit values had significant effects. In a multivariate model, only chronological age (OR: 1.143, *p* = 0.019, CI95%: 1.022 to 1.277), heart rate (OR: 1.099, *p* = 0.030, CI95%: 1.009 to 1.197), and Quick value (OR: 0.923, *p* = 0.037, CI95%: 0.856 to 0.995) had significant effects.

The comparison between deceased patients and survivors using propensity score matching revealed significant results in the univariate Cox regression analysis only for the Quick value (OR: 0.989, *p* = 0.040, CI95%: 0.978 to 0.999). For the comparison of deceased patients within the first 24 h using propensity score matching, the heart rate (OR: 1.018, *p* = 0.016, CI95%: 1.003 to 1.033), PTT value (OR: 1.014, *p* = 0.017, CI95% 1.003 to 1.026), and Quick value (OR: 0.975, *p* = 0.013, CI95% 0.955 to 0.995) revealed significant results. A multivariate calculation gave no significant results (Table 4).

## 4. Discussion

The aim of our study was to describe in more detail the cohort of patients who do not survive the primary in-hospital stay after TBI and DC. The rationale for this concept was the hypothesis that individuals with severe TBI necessitating DC may be categorized into distinct subgroups. This is already clear from the distinction between primary and secondary DC. Patients who do not survive the primary in-hospital stay are, according to our data, significantly chronological older, have more frequent pupillary dysfunction, and have a higher heart rate on admission. However, they do not differ from the survivors in the GCS on admission or in terms of Marshall score. After propensity matching, only the differences in heart rate remain. The study also compared patients who died within the first 24 h of the primary in-hospital stay with the survivors. Similarly, in this subgroup, the deceased patients were significantly chronological older and more likely to have pupillary dysfunction and a higher heart rate. They also more often had additional injuries. After propensity score matching, the significant difference in heart rate was also confirmed. With regard to the first laboratory values of the primary in-hospital stay, the deceased patients and the survivors differ in terms of PTT, Quick value, and platelet count, as well as hemoglobin. The differences in the Quick value and PTT were confirmed by propensity score matching.

### 4.1. Survival After Trauma

The number of studies dealing with the death of patients in the primary in-hospital stay after TBI and DC is limited [9,12,25,26,27,28,42]. Within the group of injured persons, patients with a TBI have a special role with regard to early death in terms of the severity of the injuries and their injury combinations with TBI [1]. Generally, for injured patients, three phases are described in which a patient can die after trauma. The first phase (immediate phase) is directly after the trauma at the accident site, within one hour of arrival at the hospital or any death in the emergency room. This is usually caused by non-survivable injuries. First-phase injuries have been described as representing 50% to 60% of the total over the last few decades. The second phase describes death within 24 h of arrival at the hospital. The severity of the initial trauma is considered to be the contributing factor in this case as well. However, advances in pre-hospital acute medicine are helping to transport patients to hospital. The proportion has been described as comprising 25% to 30% of the total. The late death phase occurs days to weeks after admission to hospital (>24 h). In recent decades, a considerable reduction to around 9% has been achieved here [4]. In terms of the consequences of trauma, there are, therefore, different subgroups. In the literature, the level of care and the length of the emergency room stay were discussed as determinants of early in-hospital death but could not be confirmed as solely influential [43,44,45,46]. Especially for patients after polytrauma, different influencing factors were identified, including bleeding control, the use of whole-body CT in the emergency room, state of consciousness at the accident site, trauma mechanism, and additional injuries, as well as pre-traumatic physical health status, age and sex, hemoglobin concentration on admission, and many more [1,47]. A meaningful clinical categorization with a clear reference to TBI has not yet been established.

### 4.2. Survival After TBI

There are a lot of factors that can influence the clinical course of severe TBI in neuro-intensive care treatment, like high blood glucose, high base excess, low mean arterial pressure, low partial oxygen/fraction of inspired oxygen ratio, and low serum hemoglobin, which are risk factors for poor outcomes [48].

There is no consensus on which instruments should be used to scientifically measure the outcome after severe TBI and at what point in time [49,50,51]. The simplest, but also less patient-orientated distinction, is the difference between the deceased and the survivors. Here, it is clinically important to understand why patients die of TBI. In an older neuropathological study, Clifton et al. found that 72% of those who died within 48 h of TBI had widespread homogenizing necrosis of neuron or direct brainstem lesions. In contrast, 19% of those who died after 48 h had these injury patterns. Patients with widespread homogenizing necrosis or direct brainstem injuries were significantly less likely to have intracranial hematomas. However, these results also show that the presence of brainstem injury is not the sole determinant of TBI subgroup classification and early death. Firsching et al. identified subgroups of patients in whom brainstem injury after TBI was detectable on MRI [52]. Patients with a bilateral upper pontine lesion after TBI had a 100 percent mortality rate. Patients with bilateral injuries of the lower medulla oblongata died weeks to months later due to pulmonary complications [52].

### 4.3. Survival After TBI and DC

Various independent predictors of outcome and mortality after severe TBI and DC and especially during the primary in-hospital stay after TBI and DC have been discussed in the literature. Angelini et al. conducted a systematic review on the effect of therapeutic antiplatelet therapy or anticoagulation on the outcome of patients undergoing DC. Although different diseases were considered in which DC was performed including TBI, no significant effect on functional outcome was found [53]. However, this study must be criticized for the fact that data on the 6-month outcome were not available for all patients. Juskys et al. found that the chronological age and the extent of cisternal compression were the strongest independent predictors of mortality during the primary in-hospital stay in patients with acute subdural hematoma who underwent either osteoplastic craniotomy or DC [54]. Hong et al. characterized radiographic predictors of outcome after TBI and DC [55]. In a multivariable model, these were age, postoperative hemispheric hypodensity, and postoperative effacement of the ambient and crural cistern. However, the effacement of the crural and quadrigeminal cistern and the postoperative hemispheric hypodensity were predictors of 6-months mortality [55]. If compression of the basal cisterns, e.g., by cerebral oedema, is understood as a cause of traumatic brainstem lesions in addition to the direct force of the trauma on the brainstem, then Firsching and Hong describe the same localization of a cause of early death during the primary in-hospital stay after TBI and DC. Although the GCS as well as pupillary dysfunction upon admission are generally strong indicators of long-term outcomes, our findings suggest that they may not be as effective in accurately identifying patients at risk for in-hospital mortality [56,57]. This is particularly true in view of the fact that the indication for decompression at a GCS value of 3 without reactive pupils is the subject of lively debate in the guidelines and the literature [58]. Increased heart rate was the only independent predictor according to our results. This might be the sign of the final herniation phase associated with pupillary dysfunction. Similarly to our results, heart rate has been proposed as an independent predictor for patients after TBI combined with extracranial trauma [59], indicating that the severity of the injury itself is relevant for further prognosis. Furthermore, older (chronological) age has been shown to be associated with worse outcome after [60]. In line with that, older patients with pupillary dysfunction were more common within the cohort of patients who did not survive the first 24 h. In the authors’ opinion, the significance and value of the various factors should be analyzed in a prospective approach using machine learning algorithms [61].

Our findings were compared with established prognostic models for mortality in patients with severe traumatic brain injury (TBI), particularly the IMPACT [62,63,64] and CRASH [65,66] models [67,68]. These models integrate key clinical predictors, such as age, GCS, pupillary response, and CT findings, to estimate mortality and unfavorable outcomes. In our matched cohort, the variables most strongly associated with mortality—such as chronological age—align with those emphasized in both the IMPACT and CRASH frameworks. However, we observed a residual imbalance in GCS between the treatment and control groups after matching (SMD > 0.2), which may partially explain differences in outcome. Given that GCS is one of the most influential clinical predictors in the aforementioned models, this imbalance must be acknowledged as a potential confounder. Furthermore, our findings can be viewed in light of pathophysiological models of TBI, which emphasize the impact of secondary injury mechanisms—such as intracranial hypertension, impaired cerebral perfusion, and systemic complications—on outcome. While our study did not directly assess intracranial pressure (ICP) dynamics, these processes likely contributed to the observed mortality pattern and are congruent with theoretical models of preventable secondary damage. Recent machine learning–based models, which aim to personalize prognosis using high-dimensional clinical and imaging data, have shown promising accuracy in mortality prediction [69,70]. Although our study employed conventional statistical matching, the consistency of our results with major predictors identified by both classical and modern models underlines the robustness of our findings. Taken together, our analysis confirms key components of theoretical TBI mortality models and highlights the importance of including validated prognostic variables when designing matched or observational comparisons.

### 4.4. Limitations

Outcome after trauma, after TBI, and DC after TBI is multifactorial. In our study, only a limited number of factors could be included in the data collection and, therefore, in the analysis. Due to the retrospective nature of this study, we are not in a position to make a statistically powered statement about the influence of extracranial injuries or other organ dysfunctions, such as hypoxia or acute respiratory distress syndrome (ARDS). In particular, data from the pre-hospital phase are missing in our study. This applies in particular to the GCS value at the scene of the accident, which certainly has a different significance than the GCS value in the emergency room. A higher GCS value in the emergency department will certainly be a greater selection factor than the GCS value at the accident site (e.g., in shock). The authors are aware that the influential time between trauma and surgery is a surrogate. It is not the time value, but the effects that occur during it. However, it is precisely because of the lack of these values that a further informative breakdown is not possible. Furthermore, no MRI images are available to adequately assess the pattern of cerebral injury, as MRI could not be performed in the first 24 h due to the required therapy. The study lacks pre-injury data, such as previous illnesses, the exact previous medication with dosage and last intake, as well as detailed knowledge about the intake of antiplatelet agents or anticoagulants or blood product substitution. The lack of data on pre-trauma use of anticoagulants and platelet aggregation inhibitors, in particular, limits conclusions, especially in older patients. This is because therapeutic compromise of coagulation is a significant factor in secondary hematoma expansion after trauma, for example, which could lead to the selection of older patients who frequently use anticoagulants. These data must be taken into account in prospective studies. In particular, the study lacks knowledge of biological or social age (frailty). The statements in this publication, therefore, relate primarily to treatment in the primary in-hospital stay. Finally, it must be taken into account that this retrospective study covers a long period of time. The authors assume that the surgical technique of DC has not changed, but that pre-hospital procedures and intensive care treatment algorithms have. Unfortunately, this cannot be calculated from the data.

## 5. Conclusions

Survival after TBI and DC can be divided into three subgroups. Namely survivors, those who died, and those who died within 24 h of the primary hospital admission. Chronologically older patients with lower GCS, pupillary dysfunction, and higher heart rate were more common in the cohort of patients who died within the hospital stay. Better characterization of patients who die during the primary in-hospital stay is important to better define the indications for DC. Prospective observational studies are needed.

## Figures and Tables

**Table 1:** Clinical characteristics for patients with in-hospital death and survivors.

	Total	Patients with In-Hospital Death	Discharged Patient	*p*	Propensity-Matched Control Patients (Discharged, Matching Tolerance = 0.05)	*p*
**N (%)**	223	65 (29.1)	158 (70.9)		41 (18.4)	
**Age in Years**						
Mean (SD)	49.9 (21.38)	55.2 (22.23)	47.7 (20.69)	**0.010**	57.0 (20.70)	0.800
Median (range)	52.0 (1–93)	59 (5–86)	49 (1–93)		61 (16–93)	
**Sex**						
Female N (%)	60 (26.9)	16 (24.6)	44 (27.8)	0.740	13 (31.7)	0.504
**Time Between Trauma and Surgery**						
Mean (SD)	28.8 (81.48)	12.2 (16.90)	35.2 (94.63)	0.186	28.7 (63.74)	0.559
Median (range)	7.0 (1–936)	5.8 (1–94)	7.3 (1–936)		7.62 (1–288)	
Mean (SD) without case (936 h)	23.8 (45.02)	12.2 (16.90)	28.2 (51.35)	0.211		
Median (range) without case (936 h)	7.0 (1–288)	5.8 (1–94)	7.0 (1–288)			
**Rate of pupil function disturbance, N (%)**	94 (44.5)	37 (63.8)	57 (37.3)	**<0.001**	24 (58.5)	0.676
**Glasgow Coma Score at Admission**						
Total						
Mean (SD)	5.0 (3.89)	5.1 (3.74)	5.0 (3.96)	0.581	6.2 (4.79)	0.272
Median (range)	3 (3–15)	3 (3–14)	3 (3–15)		3 (3–15)	
Eye Opening						
Mean (SD)	1.5 (1.03)	1.5 (1.02)	1.5 (1.03)	0.939	1.8 (1.23)	0.128
Median (range)	1 (1–4)	1 (1–4)	1 (1–4)		1 (1–4)	
Motor Response						
Mean (SD)	1.9 (1.81)	2.0 (1.80)	1.9 (1.81)	0.700	2.5 (2.22)	0.243
Median (range)	1 (1–6)	1 (1–6)	1 (1–6)		1 (1–6)	
Verbal Response						
Mean (SD)	1.5 (1.16)	1.5 (1.04)	1.5 (1.21)	0.863	1.9 (1.50)	0.208
Median (range)	1–5	1 (1–5)	1 (1–5)		1 (1–5)	
**Marshall Score**						
Mean (SD)	4.3 (1.26)	4.5 (1.29)	4.2 (1.24)	0.239	4.3 (1.40)	0.501
Median (range)	4 (1–6)	4 (1–6)	4 (1–6)		4 (1–6)	
**Vital Signs at Admission (Mean, SD)**						
Initial MAP (mmHg)	89.9 (35.08)	92.6 (36.75)	88.9 (34.48)	0.679	100.2 (22.20)	0.288
Initial pulse (beats/minute)	85.9 (24.76)	95.2 (32.37)	82.0 (19.68)	**<0.001**	84.9 (17.23)	**0.030**
**Length of ICU in days (mean, SD)**	12.6 (11.40)	5.4 (6.33)	15.5 (11.72)	**<0.001**	6.8 (4.10)	**0.009**
**Additional Injuries**						
Thorax	25 (11.2)	8 (12.3)	17 (10.8)	0.816	4 (9.8)	0.763
Abdomen	6 (2.7)	3 (4.6)	3 (1.9)	0.361	1 (2.4)	1.000
Extremities	18 (8.1)	5 (7.7)	13 (8.2)	1.000	7 (17.1)	0.207
Spine	17 (7.6)	7 (10.8)	10 (6.3)	0.273	2 (4.9)	0.477

**Table 2:** Clinical characteristics for patients with in-hospital deaths within 24 h and survivors.

	Patients with In-Hospital Death Within 24 h	Discharged Patient	*p*	Propensity-Matched Control Patients (Discharged, Matching Tolerance = 0.2)	*p*
**N (%)**	22 (9.9)	158 (70.9)		21 (9.4)	
**Age in Years**					
Mean (SD)	57.1 (24.65)	47.7 (20.69)	0.036	59.2 (18.50)	0.808
Median (range)	69 (16–86)	49 (1–93)		65 (20–81)	
**Sex**					
Female N (%)	6 (27.3)	44 (27.8)	1.000	6 (28.6)	1.000
**Time between Trauma and Surgery in Hours**					
Mean (SD)	6.5 (6.76)	35.2 (94.63)	0.033	31.4 (75.56)	0.357
Median (range)	4 (1–29)	7.3 (1–936)		5 (2–288)	
**Rate of pupil function disturbance, N (%)**	16 (76.2)	57 (37.3)	<0.001	14 (66.7)	0.743
**Glasgow Coma Score at Admission**					
Total					
Mean (SD)	4.2 (2.74)	5.0 (3.96)	0.914	7.1 (5.39)	0.175
Median (range)	3 (3–13)	3 (3–15)		3 (3–15)	
Eye Opening					
Mean (SD)	1.3 (0.80)	1.5 (1.03)	0.497	2.0 (1.38)	0.072
Median (range)	1 (1–4)	1 (1–4)		1 (1–4)	
Motor Response					
Mean (SD)	1.6 (1.28)	1.9 (1.81)	0.623	2.8 (2.38)	0.091
Median (range)	1 (1–5)	1 (1–6)		1 (1–6)	
Verbal Response					
Mean (SD)	1.3 (0.92)	1.5 (1.21)	0.664	2.3 (1.67)	0.071
Median (range)	1 (1–5)	1 (1–5)		1 (1–5)	
**Marshall Score**					
Mean (SD)	4.2 (1.10)	4.2 (1.24)	0.861	4.38 (1.28)	0.583
Median (range)	4 (1–6)	4 (1–6)		4 (1–6)	
Vital Signs at Admission (Mean, SD)					
Initial MAP (mmHg)	93.5 (31.26)	88.9 (34.48)	0.824	95.5 (35.69)	0.456
Initial pulse (beats/minute)	103.3 (25.84)	82.0 (19.68)	<0.001	76.7 (14.08)	**<0.001**
**Length of ICU in days (mean, SD)**	0.7 (0.46)	15.5 (11.72)	<0.001	4.6 (4.18)	**<0.001**
**Additional Injuries**					
Thorax	6 (27.3)	17 (10.8)	0.041	1 (4.8)	0.095
Abdomen	3 (13.6)	3 (1.9)	0.025	0 (0)	0.233
Extremities	3 (13.6)	13 (8.2)	0.420	3 (14.3)	1.000
Spine	4 (18.2)	10 (6.3)	0.074	1 (4.8)	0.345

**Table 3:** Binary logistic regression analysis for in-hospital death (comparison to discharged patients), only significant results were shown.

**Binary Logistic Regression Analysis for Death in the Primary In-Hospital Stay (Comparison to Discharged Patients with a Matching Tolerance of 0.01)**				
**Univariate Regression Analysis**	**Odds Ratio**	***p***	**CI: 95%**	
			**lower**	**upper**
Chronological age	1.018	0.017	1.003	1.032
Time between trauma and surgery	0.986	0.051	0.972	1.000
Pupil function disturbance	2.967	<0.001	1.584	5.560
Heart rate (beats/minute) on admission	1.023	0.001	1.009	1.037
Glucose (mg/dL) on admission	1.007	0.017	1.001	1.013
PTT (Seconds) on admission	1.039	<0.001	1.016	1.062
Quick (%) on admission	0.971	<0.001	0.958	0.985
Platelet count (giga/L) on admission	0.994	0.007	0.989	0.998
Hemoglobin (g/dL) on admission	0.820	0.005	0.714	0.941
**Multivariate Regression Analysis**	**Odds Ratio**	***p***	**CI: 95%**	
			**lower**	**upper**
Chronological age	1.045	0.013	1.010	1.082
Quick (%) on admission	0.965	0.049	0.931	1.000
**Binary Logistic Regression Analysis for Death in the Primary In-Hospital Stay within 24 h (comparison to discharged patients with a matching tolerance of 0.2)**				
**Univariate Regression Analysis**	**Odds Ratio**	***p***	**CI: 95%**	
			**lower**	**upper**
Chronological age	1.023	0.055	0.999	1.047
Additional thorax injury	3.110	0.037	1.073	9.020
Additional abdominal injury	8.158	0.014	1.536	43.324
Pupil function disturbance	5.389	0.002	1.874	15.499
Heart rate (beats/minute) on admission	1.043	<0.001	1.020	1.066
Glucose (mg/dL) on admission	1.009	0.029	1.001	1.017
PTT (Seconds) on admission	1.064	<0.001	1.033	1.096
Quick (%) on admission	0.936	<0.001	0.910	0.963
Platelet count (giga/L) on admission	0.993	0.033	0.986	0.999
Hemoglobin (g/dL) on admission	0.688	0.001	0.547	0.865
Hematocrit (%) on admission	0.897	0.017	0.821	0.981
**Multivariate Regression Analysis**	**Odds Ratio**	***p***	**CI: 95%**	
			**lower**	**upper**
Chronological age	1.143	0.019	1.022	1.277
Heart rate (beats/minute) on admission	1.099	0.030	1.009	1.197
Quick (%) on admission	0.923	0.037	0.856	0.995

**Table 4:** Conditional logistic regression analysis for in-hospital death (comparison to propensity score-matched discharged patients), Cox regression.

**Conditional Logistic Regression Analysis for Death in the Primary In-Hospital Stay (Comparison to Discharged Patients with a Matching Tolerance of 0.01)**				
**Univariate Regression Analysis**	**Odds Ratio**	***p***	**CI: 95%**	
			**lower**	**upper**
Time between trauma and surgery (hours)	0.992	0.201	0.980	1.004
GCS eye opening	0.880	0.311	0.687	1.127
GCS motor	0.949	0.459	0.825	1.091
GCS verbal	0.858	0.204	0.678	1.086
Additional thorax injury	1.015	0.969	0.484	2.127
Additional abdominal injury	1.677	0.382	0.527	5.344
Additional injuries of extremities	0.727	0.494	0.292	1.811
Additional spine injury	1.494	0.316	0.682	3.272
Mean arterial pressure (mmHg)	0.999	0.814	0.992	1.007
Heart rate (beats/minute) on admission	1.004	0.334	0.996	1.013
Glucose (mg/dL) on admission	1.004	0.136	0.999	1.010
PTT (seconds) on admission	1.010	0.052	1.000	1.020
Quick (%) on admission	**0.989**	**0.040**	**0.978**	**0.999**
Platelet count (giga/L) on admission	0.999	0.439	0.995	1.002
Fibrinogen (mg/dL) on admission	1.007	0.472	0.988	1.026
Leukocytes (per µL) on admission	1.017	0.212	0.990	1.045
Hemoglobin (g/dL) on admission	0.938	0.240	0.843	1.044
Hematocrit (%) on admission	0.983	0.477	0.939	1.030
**Conditional Logistic Regression Analysis for Death in the Primary In-Hospital Stay within 24 h (comparison to discharged patients with a matching tolerance of 0.2)**				
**Univariate Regression Analysis**	**Odds Ratio**	***p***	**CI: 95%**	
			**lower**	**upper**
Time between trauma and surgery (hours)	0.985	0.457	0.948	1.024
GCS eye opening	0.696	0.184	0.408	1.187
GCS motor	0.803	0.161	0.591	1.091
GCS verbal	0.698	0.148	0.429	1.136
Additional thorax injury	1.929	0.170	0.755	4.929
Additional abdominal injury	2.106	0.231	0.623	7.115
Additional injuries of extremities	0.974	0.966	0.288	3.290
Additional spine injury	1.689	0.343	0.572	4.990
Mean arterial pressure (mmHg)	0.999	0.891	0.985	1.013
Heart rate (beats/minute)	**1.018**	**0.016**	**1.003**	**1.033**
Glucose (mg/dL) on admission	1.004	0.374	0.995	1.014
PTT (seconds) on admission	**1.014**	**0.017**	**1.003**	**1.026**
Quick (%) on admission	**0.975**	**0.013**	**0.955**	**0.995**
Platelet count (giga/L) on admission	0.998	0.444	0.993	1.003
Fibrinogen on admission	1.011	0.490	0.979	1.044
Leukocytes on admission	1.015	0.463	0.976	1.056
Hemoglobin on admission	0.874	0.132	0.733	1.042
Hematocrit (%) on admission	0.948	0.129	0.884	1.016

## Data Availability

The raw data supporting the conclusions of this article will be made available by the authors on request.

## Supplementary Materials

The following supporting information can be downloaded at: https://www.mdpi.com/article/10.3390/jcm14155540/s1. Supplement Table S1: Standardized mean difference after propensity score matching.

## Abbreviations

The following abbreviations are used in this manuscript:

ANOVA	Analysis of variance
ARDS	Acquired respiratory distress syndrome
CT	Computed tomography
DC	Decompressive craniectomy
GCS	Glasgow Coma Scale
Hb	Hemoglobin
ICP	Intracranial pressure
MAP	Mean arterial pressure
MRI	Magnet resonance imaging
OR	Odds ratio
PSM	Propensity score matching
PTT	Partial thromboplastin time
SD	Standard deviation
TBI	Traumatic brain injury
