# Supplementary material for: Mortality During In-Hospital Stay and the First 24 h After Decompressive Craniectomy in Severe Traumatic Brain Injury: A Multi-Center, Retrospective Propensity Score-Matched Study
Source: J Clin Med. 2025 Aug 6;14(15):5540. doi: 10.3390/jcm14155540 (PMC12347506; doi:10.3390/jcm14155540)
Supplement: Supplementary file 1 [file jcm-14-05540-s001.zip › jcm-3740195-supplementary.pdf]

Supplement Table S1

Standardized mean difference after propensity score matching

	In-hospital deaths N=41, matching tolerance = 0.05	Deaths within first 24 hours N=22, matching tolerance = 0.2
chronological age	0.07961	0.09454
sex	0.154567	0.043524
Glasgow Coma Scale Score	0.2723	0.67142
pupil function disturbance	0.101616	0.29015
Marshall score	0.103915	0.16748
